# Malrotation Induced Small Intestine Ischemia in an Adolescent

**DOI:** 10.1155/2017/4809406

**Published:** 2017-07-31

**Authors:** Karadeniz Erdem, Atamanalp Selçuk Sabri

**Affiliations:** Department of General Surgery, Ataturk University Faculty of Medicine, Erzurum, Turkey

## Abstract

Intestinal malrotation occurs if midgut does not complete or partially completes its 270° counter-clockwise rotation around the superior mesenteric artery during embryologic life. In general, it frequently manifests with vomiting due to duodenal obstruction and volvulus in the initial months of life, and it is very rare to manifest in the adulthood. A 20-year-old male patient who had severe abdominal pain, nausea, vomiting, and distention for one day was evaluated at the emergency department. On abdominal tomography “swirling appearance of structures around the superior mesenteric artery” was reported. CT appearance was considered compatible with a rotational anomaly. Emergency surgery was planned for the patient. In laparotomy, it was observed that an approximately 100 cm long small intestine segment was rotated around a band (Ladd) and ischemia was developed in this segment due to rotation of its mesentery. The rotation of the small intestinal mesentery was corrected by opening the bands. After the warm application to the intestinal mesenteric ischemia for a while, the color and the peristalsis of the intestines became normal. The patient was discharged on postoperative day 2 with suggestions.

## 1. Introduction

Intestinal malrotation occurs if midgut does not complete or partially completes its 270° counter-clockwise rotation around the superior mesenteric artery during embryologic life [1]. Treitz ligament is not formed in these patients, and distal duodenum and jejunum are located on the right side of the vertebral column [2]. In general, it frequently manifests with vomiting due to duodenal obstruction and volvulus in the initial months of life, and it is very rare to manifest in the adulthood [3]. It manifests in adults with intermittent colic and bilious vomiting [4]. Its frequency in males and females is almost the same [5]. In acute symptomatic patients, small intestinal obstruction or ischemia is seen due to midgut or caecum volvulus, while chronic patients suffer from ambiguous abdominal pain. Peritoneal bands, which were first described by Ladd in 1932, cause these symptoms [3].

In this case, we present a 19-year-old patient who had acute abdomen and had small intestinal ischemia due to volvulus and discuss the relevant literature.

## 2. Case Presentation

**Figure 1:**
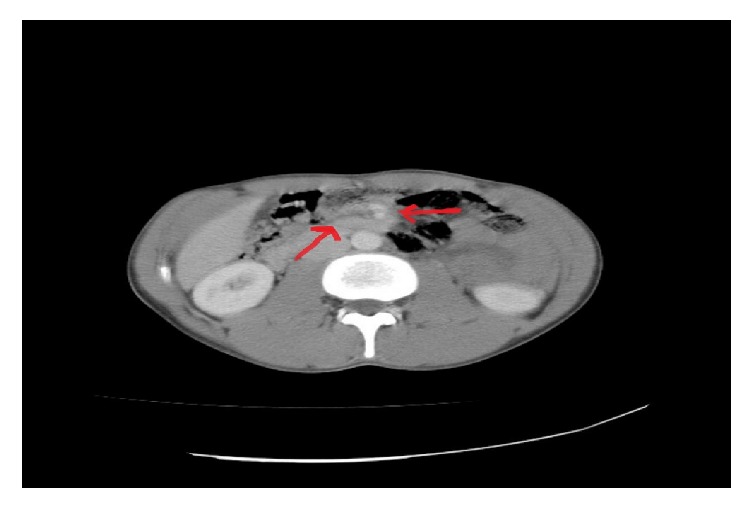
Axial contrast enhanced CT showing characteristic whirlpool sign. The structure between the two arrows shows whirlpool sign.

A 20-year-old male patient who had severe abdominal pain, nausea, vomiting, and distention for one day was evaluated at the emergency department. In patient's history, he said that he had recurrent abdominal pain and dyspeptic complaints and he has applied to the hospital for these complaints before. On the physical examination, the abdomen was distended, and there was generalized tenderness in the abdomen. Rectal examination was nonsignificant. There was not any significant finding except high white blood count (15400/mm^3^) in laboratory tests. The erect abdominal direct graph was noncontributory. On abdominal tomography “swirling appearance of structures around the superior mesenteric artery” was reported (Figure 1). CT appearance was considered compatible with a rotational anomaly. Emergency surgery was planned for the patient.

**Figure 2:**
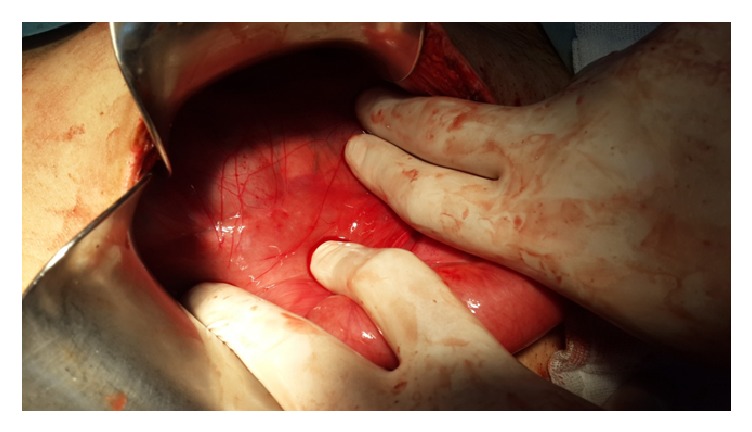
Retroperitoneal localized small intestines.

**Figure 3:**
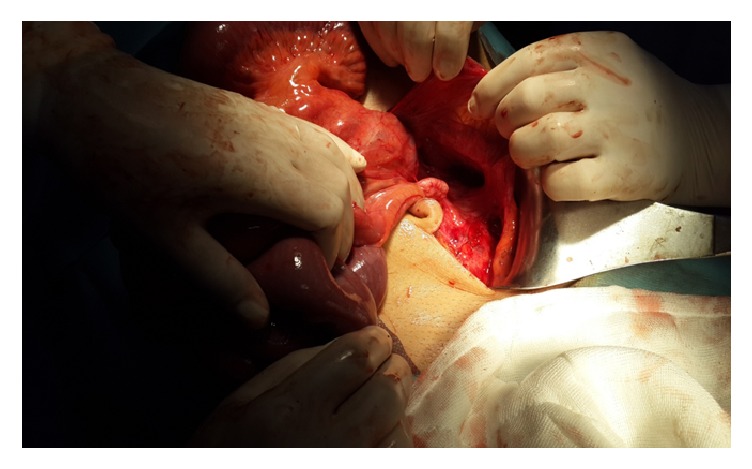
Opened peritoneal sheet.

**Figure 4:**
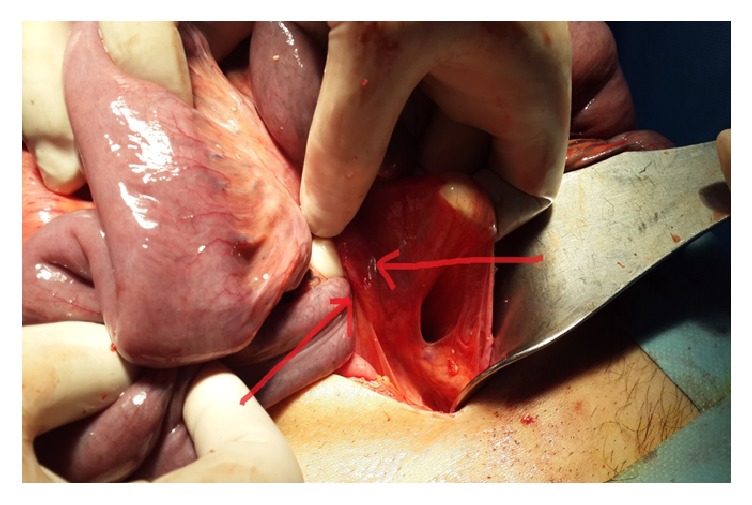
Ladd's band. The structure between the two arrows shows Ladd's band.

**Figure 5:**
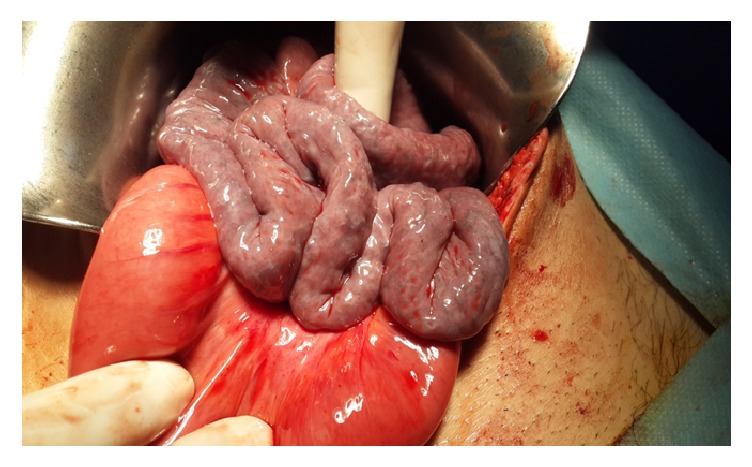
Ischemic small intestine segment.

In laparotomy, all small intestines from 80 cm proximal of the ileocecal valve were localized in retroperitoneal (Figure 2). This peritoneum was opened, and the small intestines were observed (Figure 3). There were congenital (Ladd's) bands extending from the right colon to the duodenum, exerting partial pressure to the duodenum (Figure 4). Also, the duodenojejunal junction was located on the right side of the vertebral column. In further exploration, it was observed that an approximately 100 cm long small intestine segment was rotated around a band (Ladd) and ischemia was developed in this segment due to rotation of its mesentery (Figure 5). The rotation of the small intestinal mesentery was corrected by opening the bands. After the warm application to the intestinal mesenteric ischemia for a while, the color and the peristalsis of the intestines became normal. The patient was discharged on postoperative day 2 with suggestions.

## 3. Discussion

Midgut volvulus is a serious anomaly that causes ischemia and necrosis of the small intestine due to clockwise torsion of intestines around superior mesenteric artery by adhesion of midgut mesentery as a result of rotational anomaly [6]. Most of the cases with malrotation are seen in the first month of life but, although rarely, it can also be seen in adults [2]. While most of the adult patients are symptomatic, diagnosis of malrotation is difficult since the symptoms are atypical and nonspecific. Due to the atypical symptoms, many patients with malrotation are misdiagnosed and receive malpractice [7, 8]. Although clinical findings in adults are ambiguous, patients apply with recurrent abdominal pain and vomiting probably due to partial obstruction [9, 10]. Malabsorption due to protein loss induced by low food consumption and diarrhea caused by chronic volvulus can be seen in some patients [1]. Our patient had recurrent abdominal pain and dyspeptic complaints. He did not receive a proper diagnosis at clinics where he was admitted with these complaints. When he applied to our clinic, he had small intestinal ischemia and acute abdomen related to volvulus.

The diagnosis of intestinal malrotation requires appropriate history taking, physical examination, and radiological imaging. On supine X-ray of upper GIS barium studies, the observation of duodenojejunal junction in an abnormal position rather than in its normal position (lateral of the left pedicle of the vertebra and at the same level or above the duodenal bulb) is the most specific finding of malrotation [11]. On colored Doppler USG, the observation of superior mesenteric vein (SMV) at the left rather than right of the superior mesenteric artery (SMA) should suggest malrotation. On barium colon graph, cecum can be abnormally located but seeing cecum in its normal position does not necessarily rule out malrotation. Because cecum is in its normal position (in the right lower abdominal quadrant) in 10–15% of the patients, midgut volvulus is a complication of malrotation and an emergency situation. It causes obstruction due to rotation of intestines, which are attached with short mesentery, around SMA. The rotation of duodenum, proximal jejunum, and SMV around SMA form corkscrew sign on barium graph and whirlpool sign on color Doppler USG. These findings are objective and diagnostic. They occur as a result of rotation of SMV and mesentery around SMA [12]. Whirlpool sign at superior mesenteric artery root, abnormal positioning of artery and vein, duodenal distention, and distal segmental collapse are important clues for malrotation on computerized tomography [9, 13]. There was a whirlpool appearance on the contrast enhanced abdominal computerized tomography of our patient. Also, the cecum was in normal position.

Ladd's procedure is the elective surgical treatment of intestinal malrotation since its first description in 1936 [14]. In this procedure, peritoneal bands (duodenocolic, duodenojejunal, and ileocolic) are excised, and duodenum is freed. Eventually, the small intestine is located in the right abdominal cavity and the ileocecal region is located in the left abdominal cavity [15]. Another well-known complication of the procedure is volvulus. To prevent this, cecum should be fixed, and duodenopexy should be performed after Ladd's procedure [16]. If there is an acute appendicitis suspicion at the same time, appendectomy should be added. Another indication of appendectomy is the possibility of future diagnostic challenges due to positional changes of intraabdominal organs [13]. Classical Ladd procedure was performed in our patient. Congenital bands were excised, and ischemic small intestine torsion was corrected with detorsion. Since the cecum is in the normal position, fixation was not performed. Appendectomy was performed during the surgery because the patient's appendix was edematous.

In conclusion, intestinal malrotation in adults, although rare, is an important anomaly since its complication may require emergency surgical intervention. Although its diagnosis in adults is difficult due to atypical presentation, intestinal malrotation should be considered in the differential diagnosis of patients with recurrent abdominal pain, intestinal obstruction, and dyspeptic complaints.
